# Phosphate-related genomic islands as drivers of environmental adaptation in the streamlined marine alphaproteobacterial HIMB59

**DOI:** 10.1128/msystems.00898-23

**Published:** 2023-12-06

**Authors:** Carmen Molina-Pardines, Jose M. Haro-Moreno, Mario López-Pérez

**Affiliations:** 1 Evolutionary Genomics Group, División de Microbiología, Universidad Miguel Hernández, San Juan, Alicante, Spain; University of Hawaii at Manoa, Kaneohe, Hawaii, USA

**Keywords:** HIMB59, phosphate, AEGEAN-169, phosphonate, SAR11, metagenomic islands, PstSCAB

## Abstract

Prokaryotic species are composed of multiple clonal lineages coexisting in the same habitat. In recent years, understanding the maintenance of this high intraspecific genetic diversity in asexual microorganisms has been a challenge for microbial ecology. In this study, we investigated the potential ecological role of this genomic heterogeneity in the marine HIMB59 clade. The metagenomic recruitment revealed the presence of three main genomospecies with different ecological distribution patterns within the two defined families. Genomic analysis revealed the presence of a flexible genomic island conserved throughout the order at the same position in the genome and related to phosphate (P) metabolism. The different versions of this island showed a distribution correlated with the concentration of P in the environment but not with the phylogeny at the genomospecies level. At high P availability (>0.5 µM), HIMB59 cells had only the high-affinity phosphate transporter operon (PstSCAB and PhoU). Under conditions of higher P scarcity (<0.05 µM), the cells presented a higher number of genes for the acquisition of P groups from other sources such as organic molecules and their storage. Additionally, in oligotrophic regions exhibiting extreme P depletion, such as the Mediterranean Sea, we found a second flexible genomic island related to phosphonate catabolism to supply metabolic P requirements. These results suggest that these microbes maintain in natural populations a gene pool with an equivalent biological function that allows them to respond to variations in certain micronutrients, creating ecologically distinct lineages within each species.

**IMPORTANCE:**

These results shed light on the evolutionary strategies of microbes with streamlined genomes to adapt and survive in the oligotrophic conditions that dominate the surface waters of the global ocean. At the individual level, these microbes have been subjected to evolutionary constraints that have led to a more efficient use of nutrients, removing non-essential genes named as “streamlining theory.” However, at the population level, they conserve a highly diverse gene pool in flexible genomic islands resulting in polyclonal populations on the same genomic background as an evolutionary response to environmental pressures. Localization of these islands at equivalent positions in the genome facilitates horizontal transfer between clonal lineages. This high level of environmental genomic heterogeneity could explain their cosmopolitan distribution. In the case of the order HIMB59 within the class *Alphaproteobacteria*, two factors exert evolutionary pressure and determine this intraspecific diversity: phages and the concentration of P in the environment.

## INTRODUCTION

In recent decades, our understanding of the incredible complexity, abundance, and diversity of natural microbial populations has been greatly enhanced by the development of high-throughput DNA sequencing and the advent of cultivation-independent techniques, i.e., metagenomics and single-cell sequencing. Genomic analysis of environmental prokaryotic communities revealed that microbial species are formed by consortia of different clonal lineages or subpopulations coexisting in the same habitat (1–3). These populations share the same genomic background (core genome) and a set of different flexible genes, which vary among strains within each species, acquired by horizontal gene transfer (HGT) through illegitimate or homologous recombination (4, 5). This great genetic diversity in prokaryotic communities, underestimated by culture-dependent methods, exceeds that of eukaryotes by several orders of magnitude, making them an essential part of the machinery that sustains marine ecosystems, driving the main biogeochemical cycles (6). However, the coexistence of these high levels of genomic heterogeneity within marine prokaryotic populations inhabiting offshore marine waters characterized by their oligotrophic nature is a still puzzling contradiction for microbial ecology (7). This paradox, which is contrary to the rules of the ecological principle of competitive exclusion (8), is known as the “paradox of the plankton” (9).

The HIMB59 clade, with only two representatives obtained in pure culture (10, 11), was originally described as clade V within the order *Pelagibacterales* (SAR11 clade) (10). However, several phylogenomic studies (12–14), as well as genomic classification based on the Genome Taxonomy Database (GTDB) (15), have classified these genomes as a different order to the *Pelagibacterales*. Before obtaining the first pure culture, the first sequences obtained from DNA-based clone libraries (16S rRNA genes) designated the group as AEGEAN-169 marine group (16). Amplicon-based studies of aquatic environments have found this clade to be one of the most cosmopolitan and abundant groups of pelagic bacteria inhabiting the surface of the ocean (17–22). Despite their streamlined genome, members of this order have shown great ecological flexibility, being also found in mesopelagic (18, 23) and coastal areas (24, 25) as well as associated with phytoplankton blooms (17).

The presence of only two pure cultures (10, 11), and the low recovery from metagenomic data sets of members of this order as metagenome-assembled genomes (MAGs), has hindered any in-depth study at the genomic level to understand the relationship between genetic diversity and ecology within this group. However, the number has recently increased with more than 200 genomes coming from single-cell genomics (26). This global study of the microbiome associated with the epipelagic zone of the ocean revealed that this lineage accounts for almost 5% of the bacterioplankton in the samples analyzed (26). In this study, we have used the large metagenomic data sets available in public databases together with the increase of genomes of the order HIMB59 to investigate the relationship between genomic heterogeneity and ecological distribution using a metagenomic approach. For this purpose, we recovered all the genomic diversity of HIMB59 to define ecogenomic units of classification (genomospecies), i.e., closely related phylogenomic groups with similar ecological distribution patterns (27–30). Most of the genomes could be grouped into three genomospecies with differential distribution patterns. Analysis of the genetic diversity associated with these genomospecies revealed the presence of several versions of an environmental genomic island related to P metabolism, geographically linked to its environmental concentration in the open ocean. This island was located at equivalent positions in the genome throughout the order, which would facilitate horizontal gene transfer (HGT) between population members. In addition, a second metagenomic island for phosphonate (Phn) utilization was also detected in a small subset of genomes, and its presence was limited to regions with low P concentration (Mediterranean Sea and the North Atlantic subtropical gyre). This study provides new insights into how environmental conditions (biotic and abiotic) impose evolutionary pressure for the selection of specific genes within each niche, increasing genomic diversity and creating ecologically differentiable subpopulations within each species.

## RESULTS

### Phylogenomics and ecological distribution patterns of the HIMB59 order

**Fig 1:**
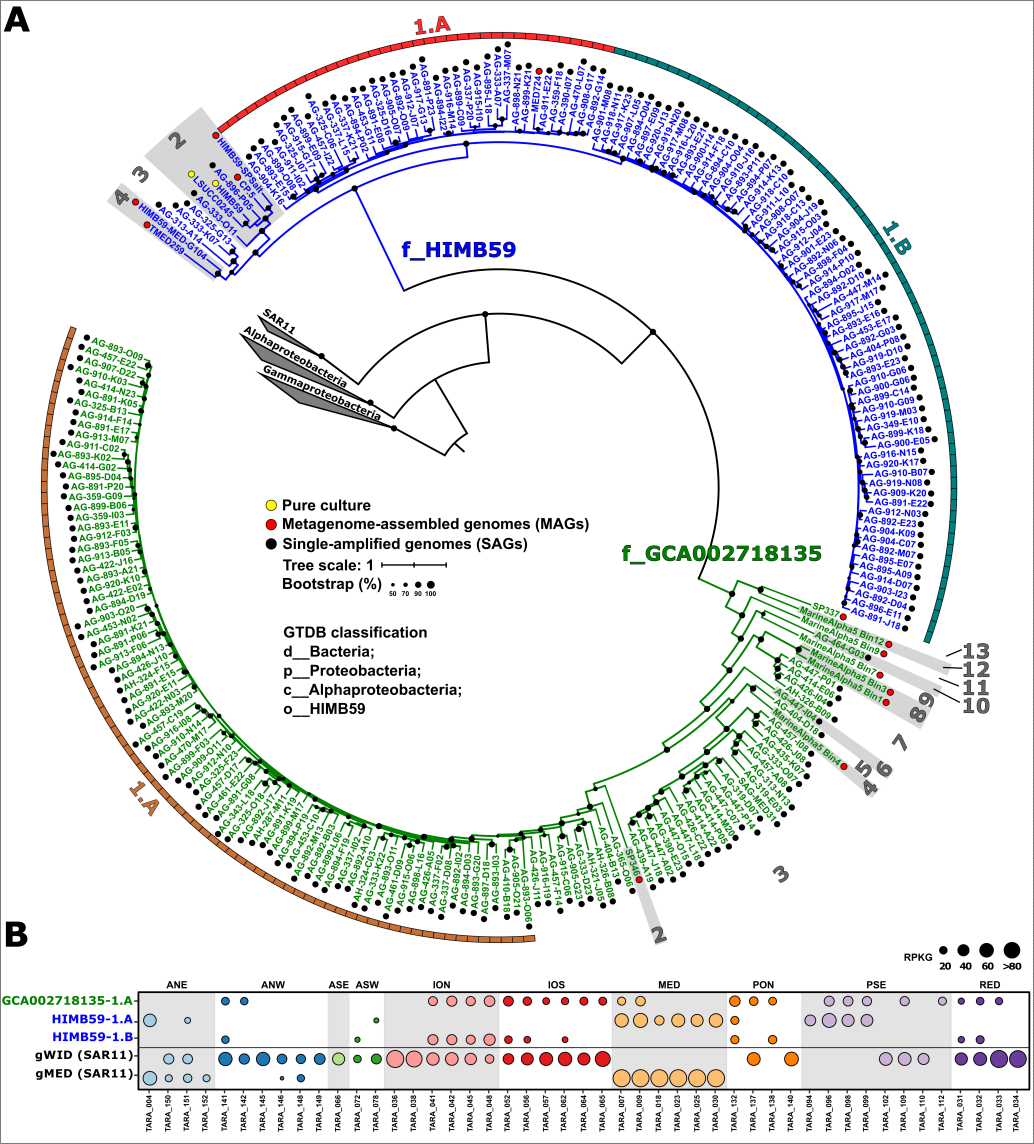
(**A**) Maximum likelihood phylogenomic tree of 243 dereplicated HIMB59 genomes (>99% ANI). The origin of the genome is indicated by colored dots next to the genome, that is MAG (red), SAG (black), or pure culture (yellow). Branches of the tree were colored according to the family classification. Gray numbers represent the genera within each family, and the three main genomospecies are highlighted with different colored squares. (**B**) Abundance (measured in RPKG) of HIMB59 genomospecies in *Tara* Oceans metagenomes compared to previously defined *Pelagibacterales* (SAR11) genomospecies. Circles are colored according to the biogeographic provinces of the *Tara* Oceans expedition. *Tara* stations are indicated on the *x*-axis. ANE, Atlantic Ocean (North East); ANW, Atlantic Ocean (North West); ASE, Atlantic Ocean (South East); ASW, Atlantic Ocean (South West); ION, Indian Ocean (North); IOS, Indian Ocean (South); MED, Mediterranean Sea; PON, Pacific Ocean (North); PSE, Pacific Ocean (South East); RED, Red Sea.

To collect as much genomic diversity as possible within the HIMB59 order, we gathered all publicly available genomes (mostly single-amplified genomes [SAGs], 2 pure cultures, and 22 MAGs) (10, 11, 13, 19, 26, 27, 31, 32) according to the GTDB taxonomic classification (Release 07-RS207) (15). Additionally, one MAG (HIMB59-SPSalt) was included in this study reconstructed from a metagenome collected in a solar saltern pond with 6% of salinity in Santa Pola (Alicante, Spain) (33). Phylogenomic analysis was carried out only on medium- to high-quality draft genomes (34) that were subsequently dereplicated (ANI > 99%) to avoid redundancies (see Materials and Methods) (35). The resulting tree clustered the final set of 243 genomes (Table S1) into two major groups classified as different families (f_HIMB59 and f_GCA002718135) based on the GTDB (15) (Fig. 1A). In addition, pairwise analysis of average nucleotide identity (ANI) (Table S2) revealed several genera within each family, named with numbers for simplicity (Fig. 1A; Table S1). The two pure cultures obtained to date were grouped in the same genus within the HIMB59 family (ANI 94.25%) (Fig. 1A; Tables S1 and S2).

Then, we used SAGs to compare genomic features between the two families. We found a statistically significant variation in the estimated genome size (*P*-value < 0.05). Members of the GCA002718135 family showed an average genome size of 1.70 Mb (standard deviation [SD], ±0.17), larger than genomes within the HIMB59 family (mean 1.47 Mb [SD,  ±0.13]) (Table S1). However, other genomic parameters such as GC content (mean GCA002718135 family 29.09% [SD, ±0.49] versus mean HIMB59 family 30.72% [SD, ±0.41]; *P-*value 0.06) and the intergenic spacer length (mean GCA002718135 family 3.32 bp ± [SD, 1.16] versus mean HIMB59 family 2.35 bp [SD, ±0.74]; *P-*value 0.12) showed no significant variation (Table S1).

The combination of the distribution in metagenomic samples (Table S3) and ANI-based pairwise similarity analysis (Table S2) of all genomes allowed the identification of seven ecogenomic units previously defined as genomospecies (Table S1) (27–30, 36). Genome recruitment showed that the entire order is preferentially found in surface waters, with only one genomospecies (GCA002718135-8.A) associated with the mesopelagic zone (>200 m deep). Three of the seven genomospecies (HIMB59-1.A, HIMB59-1.B, and GCA002718135-1.A) accounted for ca. 75% (186 out of 243) of the genomes and were selected for further studies because of their abundance (Fig. 1A; Table S1). Neither of the two pure cultures, belonging to the same phylogenomic cluster (Fig. 1A), showed enough recruitment in the metagenomic samples analyzed to be classified as a genomospecies (Table S3). Using as a reference the oceanic provinces defined in the *Tara* Oceans expedition (37), we found that the three genomospecies showed different ecological distribution patterns. While HIMB59 family genomospecies were more abundant in specific regions such as the Mediterranean Sea and the southeast Pacific for HIMB59-1.A and the north Indian Ocean for HIMB59-1.B, the GCA002718135 family genomospecies showed a more widespread distribution (Fig. 1B; Table S3). Although GCA002718135-1.A was present in a larger number of stations, the highest mean recruitment value was found in the HIMB59-1.A genomospecies (median RPKG [number of recruited reads per kb of genome per Gb of metagenome] 32.6) (Table S3). The classification of genomospecies as well as their distribution corroborates and extends the recent work of Getz et al. (14). The correlation in nomenclature between the groups described in both studies can be found in Table S1. A similar pattern to the distribution of HIMB59 genomospecies was found in the sister order *Pelagibacterales* (27). Within this order, the genomospecies endemic to the Mediterranean Sea (gMED) showed higher recruitment values than any other genomospecies, including the most cosmopolitan (gWID) (Fig. 1B) (27). It is noteworthy that, neither in *Pelagibacterales* nor in HIMB59 orders, the genomospecies with a wider distribution were found in the Mediterranean Sea suggesting a specific environmental selection. In the case of the order *Pelagibacterales*, it was attributed to the acquisition of genes related to Phn utilization by genomes belonging to the gMED genomospecies (27).

In order to complement the genomospecies information, we analyzed the genetic determinants that differentiated the three main genomospecies. This information, detailed in the supplementary material, not only extends the data previously obtained (14) but also puts the results in perspective by comparing them with several groups of marine microbes with which they share habitat. These results revealed an important role of galactose metabolism in all members of this order. These pathways, absent in the order *Pelagibacteriales* (SAR11), could represent a survival strategy to avoid competition for the same resources. Genomes of the order HIMB59 showed the highest proportion of genes (number of genes per Mb of genome) potentially related to membrane transport of all groups analyzed, with *Pelagibacteriales* (SAR11) having the second highest proportion. We found differences among the HIMB59 genomospecies in the abundance of transporters for certain nutrients (supplemental material). While genomospecies GCA002718135-1.A had the highest proportion of proteins potentially involved in saccharide, polyol, and lipid transport, HIMB59-1.A was enriched in transporters of phosphate and organophosphate molecules and the pure culture (HIMB59) had a higher fraction of mineral and organic ion transporters. Notably, genomospecies GCA002718135-1.A had an enrichment in glycosyl hydrolases, especially α-*N*-acetylgalactosaminidase, with the highest values among marine reference microbes, including specialists in polymeric carbohydrate degradation such as the bacterium of the phylum Bacteroidetes, *Polaribacter* sp. MED152 (38).

### Genetic diversity within HIMB59 genomospecies

We next sought to delve into the genomic heterogeneity within the three major genomospecies of the order HIMB59 by mapping metagenomic reads against a reference genome (39). Due to the absence of pure cultures and closed reference genomes, the first step was to select a representative genome among the three selected genomospecies. For the genomospecies HIMB59-1.A and GCA002718135-1.A, the SAG with the highest completeness was selected (AG-916-M14 [93.4% completeness and 0% contamination] and AG-891-K05 [98.8% completeness and 0% contamination], respectively) (Table S1). However, the presence of multiple SAGs with ANI >99% within the HIMB59-1.B genomospecies (Table S2) allowed their co-assembly to obtain a complete and circular reference genome referred to as complete composite genome (CCG) following the methodology previously used in references 29, 36 (see Materials and Methods).

**Fig 2:**
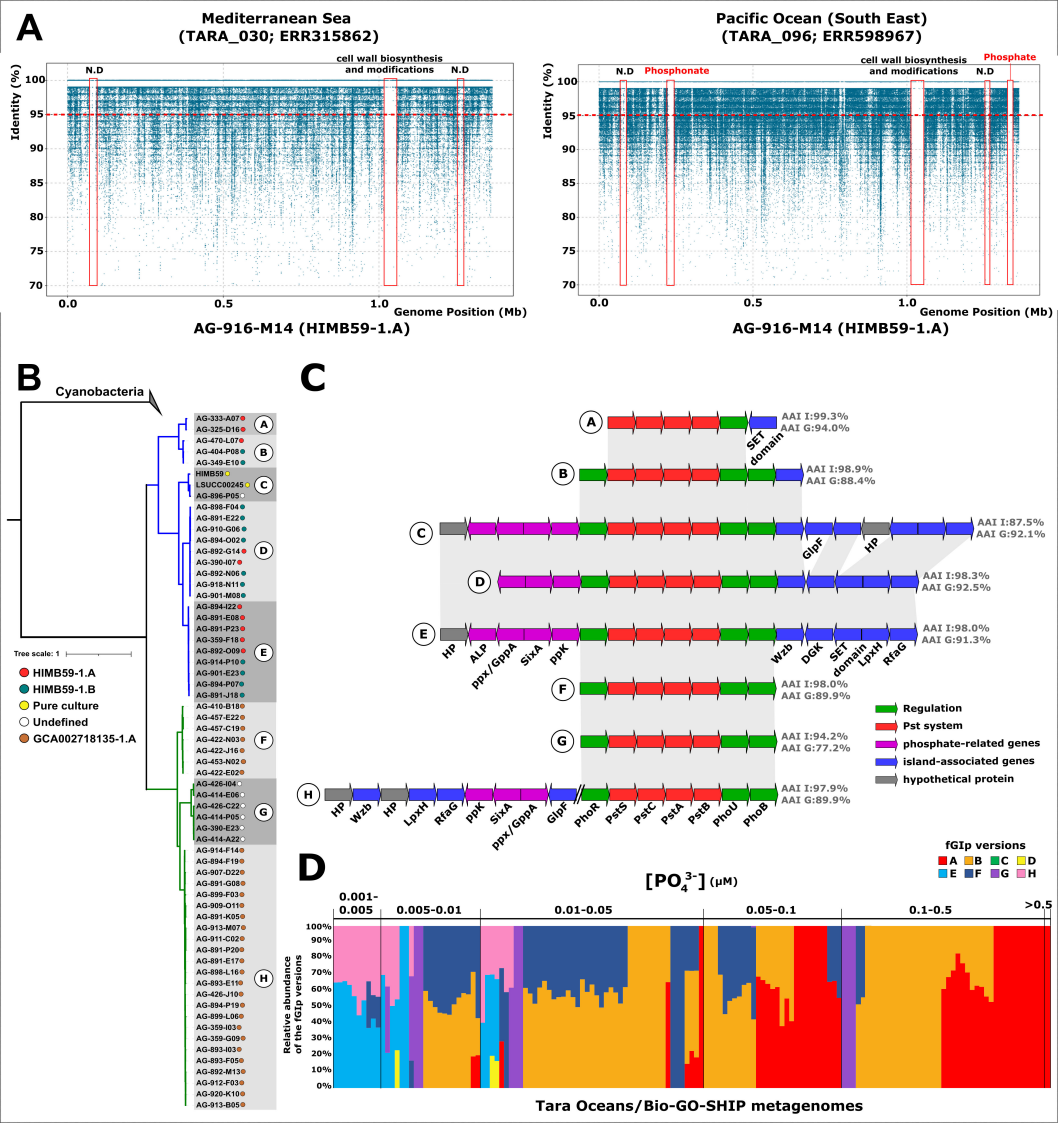
(**A**) Linear recruitment plot of HIMB59-1.A reference genome (AG-916-M14) in two metagenomes of *Tara* Oceans expedition. Each blue dot represents a metagenomic read. Metagenomic islands are highlighted in red. The red dashed line indicates the species threshold (95%). (**B**) Maximum likelihood phylogenetic tree of PstS, PstC, and PstA concatenated sequences present in SAGs of the HIMB59 order. The obtained clusters (versions) of the tree have been designated with letters (A through H). The colored dots indicate the origin of the genome, i.e., pure culture (yellow), HIMB59-1.A (red), HIMB59-1.B (green), and GCA002718135-1.A (light brown). PstACS sequences from Cyanobacteria were used as outgroup. (**C**) Genomic alignment of the flexible phosphate-related genomic islands (fGIp). At the right, the average of the amino acid identity (AAI) of the genomic island (**I**) and genome (**G**) is indicated. The two oblique lines indicate the position where the three genes belonging to the core genome are inserted in the H version. (**D**) Normalized abundance of fGIp versions in metagenomes of *Tara* Oceans and Bio-GO-SHIP expeditions. Each column represents abundance in a metagenome which are ordered according to P concentration. N.D., no determined; SET domain, methyltransferase protein domain; GlpF, glycerol uptake facilitator protein; HP, hypothetical protein; ALP, alkaline phosphatase; ppx/GppA, exopolyphosphate phosphatase/guanosine pentaphosphate phosphatase; SixA, phosphohistidine phosphatase; ppk, polyphosphate kinase; WzB, protein-tyrosine phosphatase; DGK, diacylglycerol kinase; LpxH, UDP-2,3-diacylglucosamine hydrolase; RfaG, lipopolysaccharide core biosynthesis protein; PhoR, phosphate regulon sensor protein; PhoB, phosphate regulon transcriptional regulatory protein; PstSCAB, high-affinity phosphate ABC transport system.

From the normalized metagenomic recruitment values (Table S3), linear recruitments of reference genomes were analyzed for samples in which they had a value of at least 10 RPKG with a genome coverage ≥60% and an identity threshold ≥98%. In the reference genome of the genomospecies HIMB59-1.A, we found three metagenomic islands present in all samples. The largest island was related to the lipopolysaccharide (LPS) O-chain biosynthesis, while for the remaining two metagenomic islands, no function could be inferred since they were composed mostly of hypothetical proteins (Fig. 2A). Interestingly, two other metagenomic islands appeared in a south-eastern Pacific Ocean sample (TARA_096) from the *Tara* Oceans expedition. Functional annotation of the genes revealed that these islands were related to P and Phn metabolism (Fig. 2A). In the metagenomic sample from station TARA_004, a north-eastern Atlantic Ocean station, we also located the Phn Island, but neither island was present in the Mediterranean Sea sample (TARA_030) (Fig. 2A). AG-891-K05, reference genome of the genomospecies GCA002718135-1, showed a wider distribution being present in samples from the Pacific Ocean, Indian Ocean, Red Sea, and northwest Atlantic Ocean (Table S3). Two metagenomic islands were found in all samples, one of them again related to LPS glycosylation and the other with no determined function (Fig. S1). However, recruitment in the north Indian Ocean (TARA_042), north Pacific Ocean (TARA_137), and southeast Pacific Ocean (TARA_109) revealed the presence of two new islands. In the same way as for the HIMB59-1.A genomospecies, these islands were related to P and Phn metabolism (Fig. S1). Lastly, the same pattern was maintained in the reference genome of the HIMB59-1.B genomospecies (HIMB59-1-B CCG). We found the hypervariable region related to glycotype variation (40) as well as other smaller islands. However, unlike the other two genomospecies, we found only one of the two variable metagenomic islands. While the variable P island was located in the recruitment at station TARA_042 (north Indian Ocean) but not at station TARA_141 (Northwest Atlantic Ocean), the metagenomic Phn island in this genomospecies was absent (Fig. S1). Despite genomic divergence, the border genes of the metagenomic islands of LPS, P, and Phn were equivalent throughout the order. In addition, within the HIMB59 family, the two other metagenomic islands in the reference genome of HIMB59-1.A genomospecies also had their correspondence at the same position in the reference genome of HIMB59-1.B genomospecies. Unfortunately, among all genes of these two metagenomic islands, we could only infer a BREX-like system (41) in HIMB59-1.B genomospecies related to phage defense.

### Flexible phosphate-related genomic islands

While the variation of cell envelope sugars is a universal evolutionary strategy among prokaryotes to avoid phage predation (3, 5), the presence of a gene-cassette related to P metabolism in different oceanic regions could suggest an important role of this micronutrient in the ecological adaptation to certain niches throughout the order HIMB59. Therefore, we decided to analyze the genomic diversity of this region using all the SAGs from the data set. Metagenomic recruitment allowed us to delimit the boundaries of this metagenomic island, and we were able to detect this region in 150 SAGs (61.72% of the data set) of the whole HIMB59 order. Surprisingly, genomic analysis revealed that all the islands obtained were related to P metabolism and all of them contained the *pst* operon (*pst*SCAB and *pho*U). This type of island was named flexible genomic island (fGI) following the nomenclature of previous studies to define islands present at the same position in the genome, with an equivalent biological function (5). A maximum likelihood phylogenetic tree was generated to analyze the phylogenetic diversity using a concatenation of PstSCA subunits. Sequences were clustered at 100% identity in the resulting tree for simplicity (Fig. 2B). The tree revealed two main groups that matched to the two families of the order. In addition, all sequences were clustered into eight groups, referred to with letters A to H, which did not correlate with the genomospecies classification (Fig. 2B). Analysis of additional genes associated with each group of the *pst* operon revealed that each version was characterized by a specific set of genes. Versions A through E were found in the HIMB59 family and F to H in the GCA002718135 family (Fig. 2B).

Version A (flexible phosphate-related genomic islands; fGIp-A) was the simplest of all, adding only a methyltransferase to the *pst* operon (Fig. 2C). Compared to version A, fGIp-B lost the methyltransferase, but added a tyrosine phosphatase, in addition to the two-component regulatory system PhoR-PhoB responsible for the expression of the phosphate (Pho) regulon (42). fGIp-C was found in only three genomes, two of which corresponded to pure cultures. This island is the longest of all versions with a length of 18.5 kb and a total of 19 genes. In addition to the aforementioned components such as the *pst* operon (PstSCAB and PhoU) and the two-component system PhoR-PhoB, this version included two other genes related to the Pho regulon, an alkaline phosphatase and a polyphosphate kinase. Next to the polyphosphate kinase, we found two other P-related genes such as a phosphohistidine phosphatase and an exopolyphosphatase that releases the terminal phosphate of the polyP created by the polyphosphate kinase (43) (Fig. 2C). Seven other genes were colocalized on the island, two of them related to phosphatase activity in extracellular polysaccharide formation processes, and others without any direct function related to P such as a glycosyltransferase, a diacylglycerol kinase, as well as a glycerol uptake facilitator (Fig. 2C). fGIp-D and fGIp-E were similar to version C (fGIp-C), but in version E, the glycerol uptake facilitator was lost as well as a hypothetical protein, and in version D, the Pho regulon alkaline phosphatase and another hypothetical protein were missing. The F version of the island (fGIp-F), belonging to the GCA002718135 family, contained both the *pst* operon and the two-component system PhoR-PhoB. fGIp-G is the only case where the PstSCA phylogeny did not recapitulate islands with the same gene content. We found three subtypes within this version (Fig. S2). Figure 2C shows the common part which is the same as that found in the F version. The other two subtypes added genes already mentioned in the other versions (Fig. S2). Finally, within the GCA002718135 family, the H version (fGIp-H) was the most similar to version C of the HIMB59 family. Version H showed homologs for all genes except for those coding for methyltransferase, diacylglycerol kinase, and alkaline phosphatase. In addition, we found genomic rearrangements between the two versions(C and H). Synteny was maintained for the *pst* operon, and the two-component system PhoR-PhoB, but all other genes were located upstream in the H version (Fig. 2C). Metagenomic recruitments of this version (fGIp-H) showed that three genes within the island were part of the core and, therefore, not shown in the figure. Two of them, a glycosyltransferase and a UDP-2,3-diacylglucosamine diphosphatase (LpxH), are paralogous to those in the island as well as a methyltransferase.

The pairwise comparison of the average amino acid identity (AAI) of the islands of each version was always higher than the genome-wide comparison (Wilcoxon signed-rank test, *T* = 1.0, *P*-value = 0.016), which reinforces the variable character of this region (with the only exception of version C) (Fig. 2C). In addition to these variations in the genetic content of fGIp, we searched for other Pho regulon-related genes in the genomes that contained the *pst* operon. In 55 of the 67 genomes of the GCA002718135 family, we found the low-affinity Pi transporter (Pit), considering the incomplete nature of SAGs we could say that it is an intrinsic feature of this family. Among the 83 analyzed genomes of the HIMB59 family that contained the *pst* operon, this Pit was only found in four genomes. In two of them, the gene encoding Pi was part of the core genome, and in two other genomes, it was inserted in the fGIp. Apart from fGIp, no other genes related to P metabolism were found in the genomes of the HIMB59 family.

### Ecological distribution of flexible phosphate-related genomic islands

Once the gene content of the different fGIp versions was established, we evaluated the global distribution patterns of the fGIp versions using the large data set from the *Tara* Oceans expedition (44) as well as the Bio-GO-SHIP (45) and correlating them with P concentration (Fig. 2D). In stations with the highest P concentrations (>0.5 µM), we found only the simplest version, fGIp-A, which lacks the two-component system PhoR-PhoB (Fig. 2C and D). Versions with a smaller number of auxiliary genes other than the *pst* operon and the two-component system PhoR-PhoB (fGIp-A, B for the HIMB59 family and fGIp-F, G for the GCA002718135 family) were found in stations with concentrations between 0.05 and 0.5 µM (Fig. 2D). Regarding the oceanic provinces, versions A and B had a wider distribution and could be found at stations in the Atlantic and Indian Oceans as well as the only ones present in the Pacific Ocean. In the Red Sea, we only found version F of the family GCA002718135, which was also found in the Indian and Atlantic Oceans. Version G could only be located at stations in the North Atlantic. The only exception was version C found only in pure cultures. This version, despite having the highest number of genes, did not recruit in the offshore metagenomes probably because both cultures were isolated from coastal areas. Conversely, fGIp with the highest number of genes from both families (fGIp-E for HIMB59 family and fGIp-H for GCA002718135 family) were the only ones present in the stations with the lowest P concentrations (0.001–0.005 µM) (Fig. 2D) located exclusively in the Mediterranean Sea and in the Northwest Atlantic Ocean regions.

Statistical analyses corroborated the correlation between the different variants of fGIp and P concentration (Table S4). Spearman’s rank correlation coefficients (*P*-value < 0.01) showed a strong (*ρ* = 0.59) and a weak (*ρ* = 0.24) positive correlation for variants A and B, respectively, while variants D, E, and H showed a strong negative correlation (*ρ* values of −0.6; −0.56; −0.55) (Table S4). Variant G showed a moderate negative correlation (*ρ* = −0.48) and F had a weak correlation (*ρ* = −0.16). Unfortunately, we were unable to predict any statistical value for variant C because it was not present in any sample as previously stated. Lastly, the Kruskal-Wallis *H* test (*H* = 37.37, *P*-value = 10^−6^) indicated that phosphate concentrations differed significantly among the eight fGIp variants. Therefore, these results suggest a correlation between the availability of P in the environment and the genomic variants of the island generating ecological lineages within each species adapted to different ecological niches.

### Flexible phosphonate-related genomic islands (fGIphn)

In the same way we did for fGIp, we analyzed the other metagenomic island identified, which was related to Phn metabolism, a type of phosphorus metabolite characterized by a C-P bond that constitutes more than 20% of the dissolved organic P in the ocean (46). Recently, using the same set of genomes, it was shown that while *Pelagibacterales* had the potential for phosphonate production and consumption in the surface ocean, HIMB59 were mostly consumers (47). First, we identified among the group of genomes that had the *pst* operon (#150) those that also contained the flexible phosphonate-related genomic islands (fGIphn). We found a total of 24 fGIphn (16% of the genomes), 13 of them in the HIMB59 family and 11 in the GCA002718135 family. Based on the genetic content of the fGIphn we identified four versions, versions A and B belonging to genomes of the HIMB59 family and versions C and D to the GCA002718135 family.

**Fig 3:**
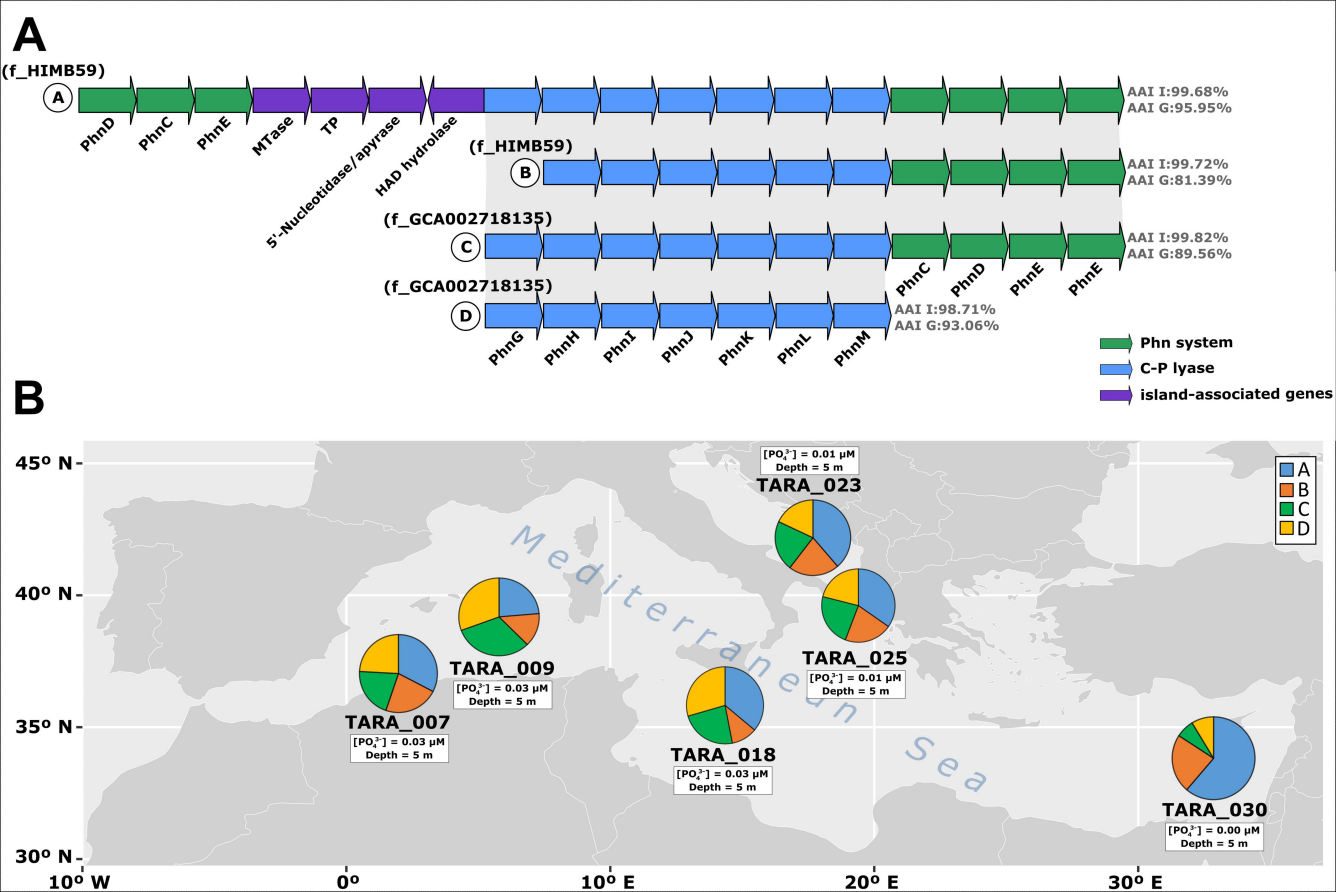
(**A**) Genomic alignment of the flexible phosphonate-related genomic islands (fGIphn). At the right, the average of the amino acid identity (AAI) of the genomic island (I) and genome (G) is indicated. (**B**) Relative abundance of each version of fGIphn (**A–D**) throughout the Mediterranean Sea. Depth and phosphate concentration are indicated for each sample. PhnD, phosphonate-binding periplasmic protein; PhnC, phosphonate import ATP-binding protein; PhnE, phosphate-import permease protein; MTase, methyltransferase; TP, transmembrane protein; HAD, haloacid dehalogenase; PhnGHIJKLM, C-P lyase genes.

Version A (fGIphn-A) was the most complete and exclusively found in the HIMB59-1.A genomospecies. We found the seven genes constituting the core C-P lyase complex (*phn*GHIJKLM) and two ABC transport systems for Phn (*phn*DCE and *phn*CDEE) located on both sides of the C-P lyase, as well as four auxiliary genes encoding a methyltransferase, a hydrolase, a transmembrane protein, and a 5′-nucleotidase/apyrase (Fig. 3A). In the B and C versions of the island (fGIphn-B and fGIphn-C), one from each family, we found only one of the ABC transporters (*phn*CDEE) and the C-P lyase operon. However, the *phn*G gene was lost in the fGIphn-B version (Fig. 3A). fGIphn-D was the only version in which no specific Phn transporter was found, only the C-P lyase operon (Fig. 3A).

Regarding the ecological distribution of fGIphn, metagenomic recruitment in the same databases mentioned above revealed that all versions were restricted to the Mediterranean Sea and the central North Atlantic gyre (Fig. 3B; Fig. S3), regions characterized as P-limited environments (48–50). Figure 3B shows the distribution of the versions within the Mediterranean Sea. The results showed a higher abundance of version A (the most complete version) in the eastern basin than in the western basin, which has even more limiting concentrations of P (50). In the same line, we could detect a similar pattern in the North Atlantic gyre, where in most of the stations analyzed, version A was the most abundant (Fig. S3). Similar to fGIp, these results suggest that the concentration of P selects certain versions of the island in the population.

## DISCUSSION

Both earlier studies based on 16S rRNA (referred to as AEGEAN-169) (16–18, 20, 26) and more recent studies using genomes from single-cell genomics (26) revealed that the order HIMB59, composed of photoheterotrophic microbes, is one of the dominant groups in the upper layers of the epipelagic ocean. Although this work is focused on the study of genetic diversity, it is worth noting that a study describing the phylogenomics, ecological distribution, and metabolic potential of the order HIMB59, as well as its relationship with the order *Pelagibacterales*, has recently been published (14). The two papers provide complementary insights into the genomic diversity and lifestyle within this order.

We took advantage of the increase in the number of genomes within the order HIMB59 and its abundance in the large metagenomic data sets available to study the high degree of genetic diversity found in marine prokaryotic populations from an ecological point of view. Alignment of metagenomic reads against a reference genome is a useful approach to trace variable areas of the genome, i.e., metagenomic islands. This approach reflects the variation in the flexible component of the genome among clonal lineages (or subpopulations) that make up a species (39). Regardless of the reference genome and metagenome, we always found an island involved in the synthesis of the glycosidic envelope of the outer part of the cells. This is a common feature observed in free-living microbes from aquatic environments and its hypervariability is related to variation in glycotypes to evade phage recognition (3, 51). The high genetic heterogeneity of this island in clones coexisting in the same sample helps to decrease phage selective pressure (3, 40). We also found other types of islands present in some metagenomes and absent in others, which could be related to the adaptation to a specific ecological niche. Genomic analysis revealed that these islands were present in all genomes, and their location in the genome was conserved. Most importantly, although the gene pool varied among genomes, the biological function was equivalent and related to P metabolism. For all these reasons, these regions were named flexible phosphate-related genomic islands (fGIp) (5).

Phosphorus is an essential nutrient for all organisms (52), needed for core physiological processes, such as phospholipids, nucleic acids, and energy conservation (e.g., ATP, NADPH biosynthesis) (53, 54). Thus, depending on the environment, it can limit the growth of marine phytoplankton (54). The main source of phosphorus for marine microorganisms is inorganic phosphate, easily obtained from the environment using the high-affinity phosphate transporter (*pst* operon), although some organisms also harbor a low-affinity phosphate transporter (Pit) (54). However, in regions with low P availability, microorganisms can also obtain phosphorus through a myriad of dissolved organic phosphorus compounds (DOP). To do that, they encode several enzymes, such as phosphoesterases and phosphohydrolases (55) that allow for the cleavage of the phosphate bond. Therefore, the enrichment of certain variants of the island with genes related to the degradation of DOP in regions with differential P concentration seems to indicate a natural selection of a set of environmental genes that allow the population to adapt to multiple ecological niches in response to different nutritional requirements. This phenomenon has already been described in some genomes of the Roseobacter lineage (56) as well as in marine cyanobacterium *Prochlorococcus* (57–59), on which a relationship between ocean environmental P concentrations and genomic content has also been observed. In the same way as we noticed for genomes of the order HIMB59, at low P concentrations, *Prochlorococcus* cells presented higher numbers of genes related to P acquisition, utilization, and regulation. As observed in HIMB59, the largest phosphate-related genomic island was found in *Prochlorococcus* MED4, a member of the high-light intensity ecotype (HL-I) isolated in the ultra-oligotrophic Mediterranean Sea (57). These islands were enriched in genes to degrade DOP. Both taxa encoded alkaline phosphatases although HIMB59 encoded a PhoX whereas *Prochlorococcus* had a PhoA. The presence of this gene has been extensively studied, as it is significantly enriched and active in P-depleted regions (55, 60–62). However, while *Prochlorococcus* MED4 only had a glyceraldehyde-3-phosphate dehydrogenase in the genomic island, HIMB59 showed a higher potential to degrade more DOP compounds, as we found a tyrosine-specific protein phosphatase (Wzb) and a UDP-2,3-diacylglucosamine diphosphatase (LpxH). The former participates in extracellular polysaccharide synthesis by dephosphorylation of Wzc, a tyrosine kinase (63), while the latter catalyzes one of the steps in lipid A biosynthesis by cleaving the pyrophosphate bond of UDP-2,3-diacylglucosamine (64). Both reactions release P that could also be utilized by the cell. Besides, HIMB59 encoded for a polyphosphate kinase (65) that allows for the storage of P in the form of linear polymers of inorganic orthophosphate (polyP). They also had an exopolyphosphatase that releases the terminal phosphate from polyP in response to phosphate and amino acid limitations (66). As somewhat expected, most of these genes were lost at concentrations higher than 0.1 µM (57, 67) where cells only encoded for the *pst* operon and the PhoR-PhoB regulator. Remarkably, in HIMB59, these regulatory proteins were absent at high P concentrations (>0.5 µM), which could indicate that they are potentially insensitive to environmental P changes. The acquisition of this two-component system together with the *pst* operon appears to be sufficient to be able to adapt to concentrations between 0.05 and 0.5 µM.

However, all these adaptations do not seem to be enough to cover the metabolic needs of P in ultra-oligotrophic regions such as the Mediterranean Sea and the North Atlantic subtropical gyre characterized by a high N:P ratio with respect to the global ocean (49, 68, 69). In these regions, where P concentrations are in the nanomolar range (70), we found a second island related to Phn catabolism, which can be used as a source of P and, under certain conditions, as a source of carbon and nitrogen (71, 72). Likewise, in the order *Pelagibacterales*, an endemic genomospecies from the Mediterranean Sea (gMED) was found (27). Comparison with other *Pelagibacterales* genomospecies, gMED revealed the specific presence of a set of genes involved in phosphonate utilization but, in this case, colocalized in the same genomic island as the P metabolism genes (*pst* operon and PhoR-PhoB) (27). Therefore, these data suggest an evolutionary convergence of these two closely related orders in their adaptation to extremely P-limited systems such as the Mediterranean Sea. As has been observed for P, we also observed a variation in the gene content of the phosphonate island throughout the Mediterranean Sea. In the Eastern basin that shows a higher oligotrophy (73), apart from the C-P lyase our data indicated a higher abundance of a Phn version with two ABC transporters (*phnDCE* and *phnCDEE*) instead of those like the B and C variants (*phnCDEE*). The presence of these two transport systems in *Prochlorococcus* was associated with the differential transport of molecules, while the transporter encoded by the *phnDCE* operon only transports phosphite and *phnCDE* could also transport simple phosphonates (methylphosphonate and ethylphosphonate) (74). Lastly, version D only harbored the C-P lyase core complex with no specific transporter associated. However, we could detect on the same island an ABC transport system that, although being tentatively annotated as oligopeptide transporter, its function may be related to Phn acquisition.

All versions of the island shared the *pst* operon, but the absence of an association to the phylogeny at the genomospecies level, as well as a higher mean AAI than the whole genome, may suggest frequent horizontal transfer. This result agrees with previous reports in *Prochlorococcus* genomes (57, 59). The absence of typical hallmarks that characterize illegitimate recombination such as the presence of IS elements, or tRNA at the boundaries of the islands, and the location of the island at the same position in the genome of all strains could indicate that the mechanism of exchange is homologous recombination (4, 5). Since the ocean surface is the ecological niche of this type of microbes in which there are no geographical barriers to genetic exchange, homologous recombination could maintain the genomic diversity of this island as suggested previously (39, 75). It is important to note, though, that most of the SAGs used in this study that had the flexible P island (117 out of 150) came from a single sample obtained from the Sargasso Sea (26). From this sample, we could recover all the different versions of the P-related genomic islands, which shows that local diversity is comparable to global diversity, which is consistent with the hypothesis “everything is everywhere, but the environment selects” (76).

In the same way that has been observed in *Pelagibacterales*, this environmental genomic diversity may help populations adapt to variations in certain micronutrients by creating different ecologically distinct lineages within each species (27, 39, 77). This characteristic provides the population with the capacity to adapt to changes in the nutritional conditions of the environment. Therefore, maintaining a pool of environmental genes (flexible genomic islands) related to an equivalent biological function within the population seems to be a good evolutionary strategy in microbes with streamlined genomes. In addition, this study highlights the importance of studying the flexible component of the genome to understand the high level of genomic heterogeneity of marine prokaryote populations, as well as the use of techniques such as single-cell genomics to obtain the full genomic potential of the population (pangenome) as opposed to the limitations of MAGs generated with second-generation sequencing (34, 78). The advent of new third-generation technologies (long read technologies) will improve, in the near future, the recovery of genomes with a higher degree of completeness including the flexible genome (79). Therefore, these results show the relationship between environmental conditions and genomic diversity within a species, as well as the relevance of the environmental gene pool of the population as an important mechanism in the evolutionary success of marine bacteria with streamlined genomes.

## MATERIALS AND METHODS

### Phylogenomic classification of HIMB59 order

All available genomes classified as HIMB59 based on the Genome Taxonomy Database (GTDB) (15) up to March 2022 (Release 07-RS207) were downloaded from the National Center for Biotechnology Information (NCBI). To refine the phylogenomic classification of HIMB59 order, we first discarded genomes with completeness <50% and contamination >5%, estimated with CheckM (80). Then, genomes were de-replicated (ANI >99%) using dRep software (35). Thus, from the 439 starting genomes, 243 were used to classify the sequences phylogenomically using Phylophlan 3.0 using the following parameters: *-*d phylophlan -t a -diversity high -accurate -f supermatrix_aa.cfg (81). Genomes belonging to *Pelagibacterales* (SAR11), and others from Alphaproteobacteria and Gammaproteobacteria classes were included as an outgroup. iTOL (82) was used for tree analysis and editing. The final tree was built using a total of 396 single-copy gene sequences with a final alignment of 36,946 amino acid positions. Family and genus classification were defined by combining GTDB classification, phylogenomic tree topology, and average nucleotide identity (ANI). ANI values among genomes were calculated using pyani (83) package with ANIblastall subcommand parameter.

### Genomic features

Prodigal v2.6.3 (84) was used to predict coding DNA sequences (CDS) that were functionally annotated using DIAMOND v2.0.6 (85) against the NCBI database of non-redundant protein sequences (NCBI nr) and using HMMscan v3.1b2 (86), for COG (update 2014) (87), and TIGFRAM v15.0 (September 2014) (88) databases.

### Metagenomic recruitment

Several publicly available metagenomes were used for recruitment of metagenomic reads against genomes to explore their ecological distribution. In particular, samples from *Tara* Oceans expedition (44), GEOTRACES (89), a Mediterranean Sea depth profile (19), and The Global Ocean Ship-based Hydrographic Investigations Program (Bio-GO-SHIP) (45). Prior to the analysis, raw reads were trimmed with Trimmomatic v0.39 (90). Then, high-quality metagenomic reads were aligned against genomes using BLASTn v2.10.1 (length ≥50 bp, identity ≥98%, *e*-value ≤1*e*^−5^) (91). The results obtained were used to compute the RPKG (reads recruited per kb of genome per Gb of metagenome) values that provide a normalized number comparable across various metagenomes (Table S3). The presence of a genome in a metagenome was determined as positive when the RPKG value was at least 10 with a genome coverage ≥60% and an identity threshold for metagenomic reads of ≥98%. For the linear recruitment of the reference genomes of each genomospecies, the BLASTn cut-off value established for the alignment of the reads was 70% nucleotide identity over a minimum alignment length of 50 nucleotides. The resulting alignments were plotted using the ggplot2 package in R.

### Genome reconstruction

The presence of genome clusters belonging to the same clonal frames (ANI >99%) for genomospecies HIMB59-1.B allowed us to co-assemble contigs to obtain a complete genome using the same approach employed previously (29, 36). Briefly, all contigs from SAGs AG-892-D04, AG-894-G11, AG-915-C07, AG-917-N20, and AG-891-G06 were reassembled using Flye v2.9 (92) with the “subassemblies” option and an expected genome size of 1.4 Mb. The obtained genome was sorted starting with the *dnaA* gene and named as HIMB59-1.B CCG (BioSample SAMN36283487).

### Metabolic analyses of reference genomes

Proteins from the three reference genomes of the main HIMB59 genomospecies, the first pure culture (HIMB59), and other representative marine microbial genomes were functionally annotated and compared against the SEED subsystem database (93) using DIAMOND v2.0.6 (85) (≥40% identity and ≥50% coverage) and KEGG (KEGG Mapper, Reconstruct Brite, KEGG Orthology) (94) through the tool BlastKOALA V.2.2 (95). More accurate annotation was done for peptidases, transporters, and glycoside hydrolases using MEROPS, KEGG, and CAZy databases respectively (94, 96, 97).

### Characterization of flexible genomic phosphate and phosphonate islands

Recovery of *pst* operon from all genomes was achieved using a custom database of *pstS*, *pstC,* and *pstA* sequences, aligning them by muscle (98) and building Hidden Markov models (HMMs) using hmmbuild, performing the screening of putative ones by hmmscan (86). For the Phn operon, the same approach was done with *phnC*, *phnD,* and *phnE* sequences in all SAGs that had *pst* operon. Finally, other genes of Pho regulon were searched in the same way, in particular, *phoU*, *phoB*, *phoR*, *pit*, *ppk,* and *ALP*. A maximum likelihood phylogenetic tree was constructed using iqtree (99) with 5000 ultrafast bootstraps and the -m MFP option to find the best model that fitted to our data.

### Statistical analyses

All statistical tests applied in the data were performed in python using the SciPy package (100). Specifically, the Spearman’s rank correlation, Kruskal-Wallis, and Wilcoxon signed-rank tests were performed with the functions stats.spearmanr, stats.kruskal, and stats.wilcoxon, respectively, with default options.

## Data Availability

The reconstructed genome HIMB59-SPSalt has been deposited as BioSample SAMN36283488 and HIMB59-1.B CCG has been deposited as BioSample SAMN36283487 under BioProject PRJNA993420 in the NCBI.

## SUPPLEMENTAL MATERIAL

Figure S1 msystems.00898-23-s0001.pdf Recruitment plot of the reference genomes for the genomospecies GCA002718135-1.A and HIMB59-1.B in two metagenomes of the Tara Oceans expedition.

Figure S2 msystems.00898-23-s0002.pdf Genomic characterization of the different subtypes of the G version of fGIp.

Figure S3 msystems.00898-23-s0003.pdf Relative abundance of each version of the flexible phosphonate-related genomic island (fGIphn) in North Atlantic Ocean samples from the Tara Oceans and Bio-GO-SHIP expeditions.

Supplemental Information msystems.00898-23-s0004.pdf Supplemental text, Fig. SI-1, and Tables SI-1 to SI-3.

Table S1 msystems.00898-23-s0005.xlsx Genomic features of HIMB59 genomes.

Table S2 msystems.00898-23-s0006.xlsx Pairwise comparison of average nucleotide identity (ANI) of the genomes of the HIMB59 clade.

Table S3 msystems.00898-23-s0007.xlsx Recruitment values (RPKGs) for all genomes at 98% identity.

Table S4 msystems.00898-23-s0008.xlsx Statistical analysis between versions of the flexible phosphate-related genomic islands and environmental P concentration.

ASM does not own the copyrights to Supplemental Material that may be linked to, or accessed through, an article. The authors have granted ASM a non-exclusive, world-wide license to publish the Supplemental Material files. Please contact the corresponding author directly for reuse.
