# Supplementary material for: Phosphate-related genomic islands as drivers of environmental adaptation in the streamlined marine alphaproteobacterial HIMB59
Source: mSystems. 2023 Dec 6;8(6):e00898-23. doi: 10.1128/msystems.00898-23 (PMC10734472; doi:10.1128/msystems.00898-23)
Supplement: Figure S1 — Recruitment plot of the reference genomes for the genomospecies GCA002718135-1.A and HIMB59-1.B in two metagenomes of the Tara Oceans expedition. [file msystems.00898-23-s0001.pdf]

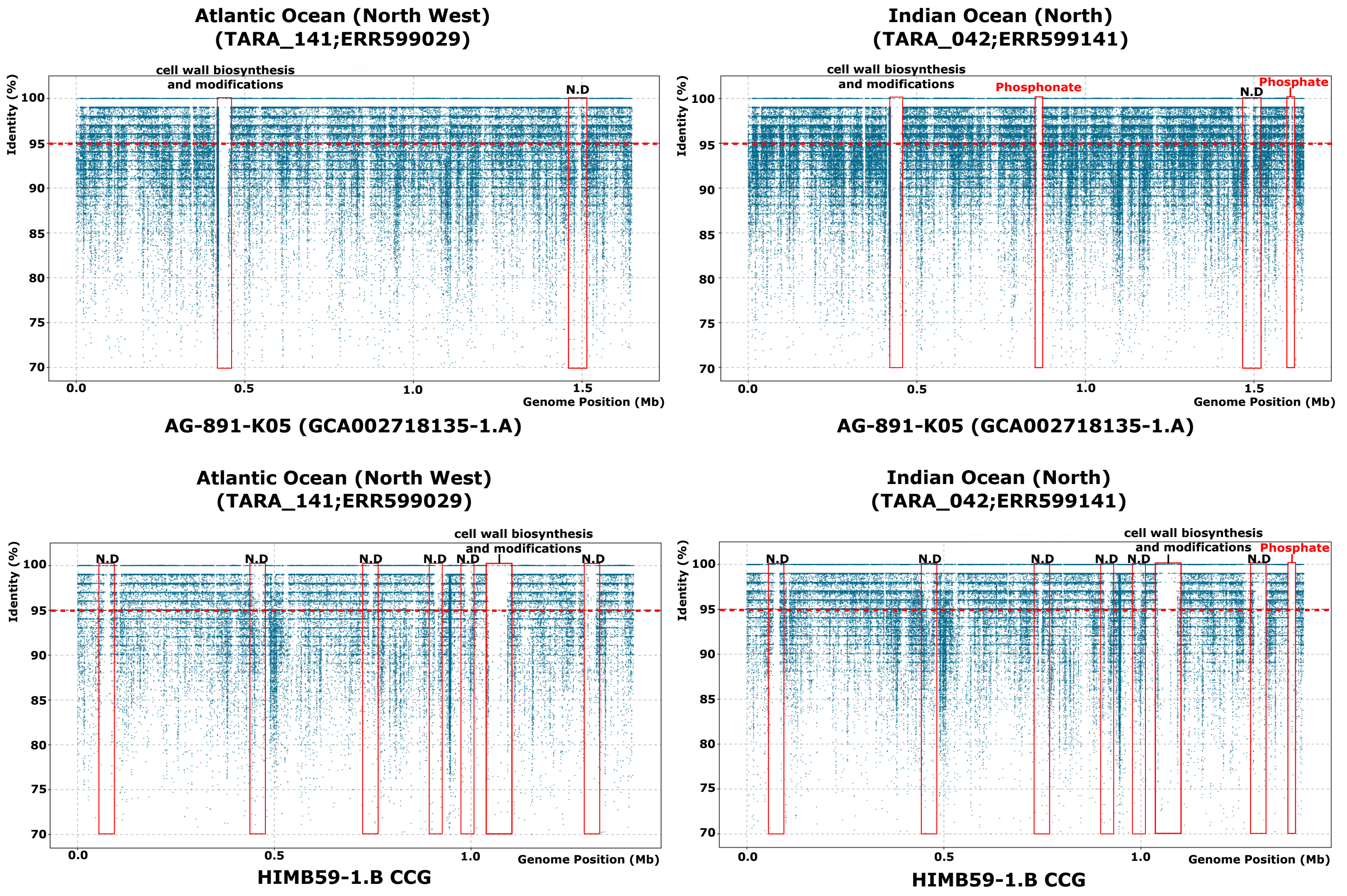

Figure S1. Recruitment plot of the reference genomes for the genomospecies GCA002718135-1.A and HIMB59-1.B in two metagenomes of the Tara Oceans expedition. Metagenomic islands are highlighted in red. The red dashed line indicates the species threshold (95%).
