# Supplementary figures and images for: Phosphate-related genomic islands as drivers of environmental adaptation in the streamlined marine alphaproteobacterial HIMB59
Source: mSystems. 2023 Dec 6;8(6):e00898-23. doi: 10.1128/msystems.00898-23 (PMC10734472; doi:10.1128/msystems.00898-23)

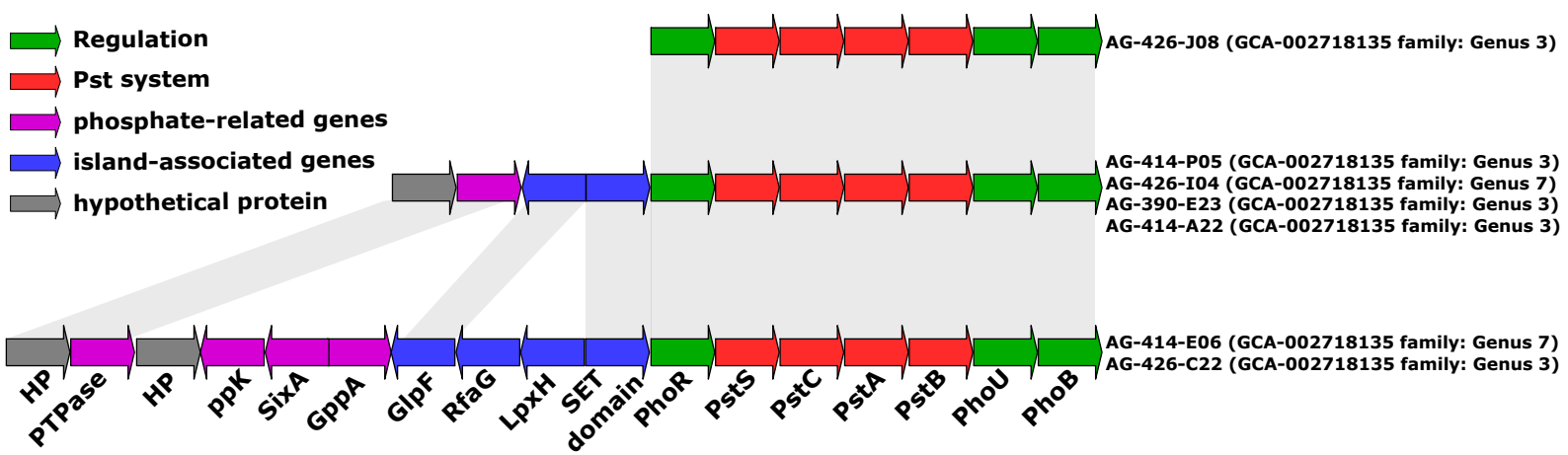

**Figure S2.** Genomic characterization of the different subtypes of the G version of fGIp.

Supplement: Figure S2 — Genomic characterization of the different subtypes of the G version of fGIp. [file msystems.00898-23-s0002.pdf]
