# Supplementary material for: Phosphate-related genomic islands as drivers of environmental adaptation in the streamlined marine alphaproteobacterial HIMB59
Source: mSystems. 2023 Dec 6;8(6):e00898-23. doi: 10.1128/msystems.00898-23 (PMC10734472; doi:10.1128/msystems.00898-23)
Supplement: Figure S3 — Relative abundance of each version of the flexible phosphonate-related genomic island (fGIphn) in North Atlantic Ocean samples from the Tara Oceans and Bio-GO-SHIP expeditions. [file msystems.00898-23-s0003.pdf]

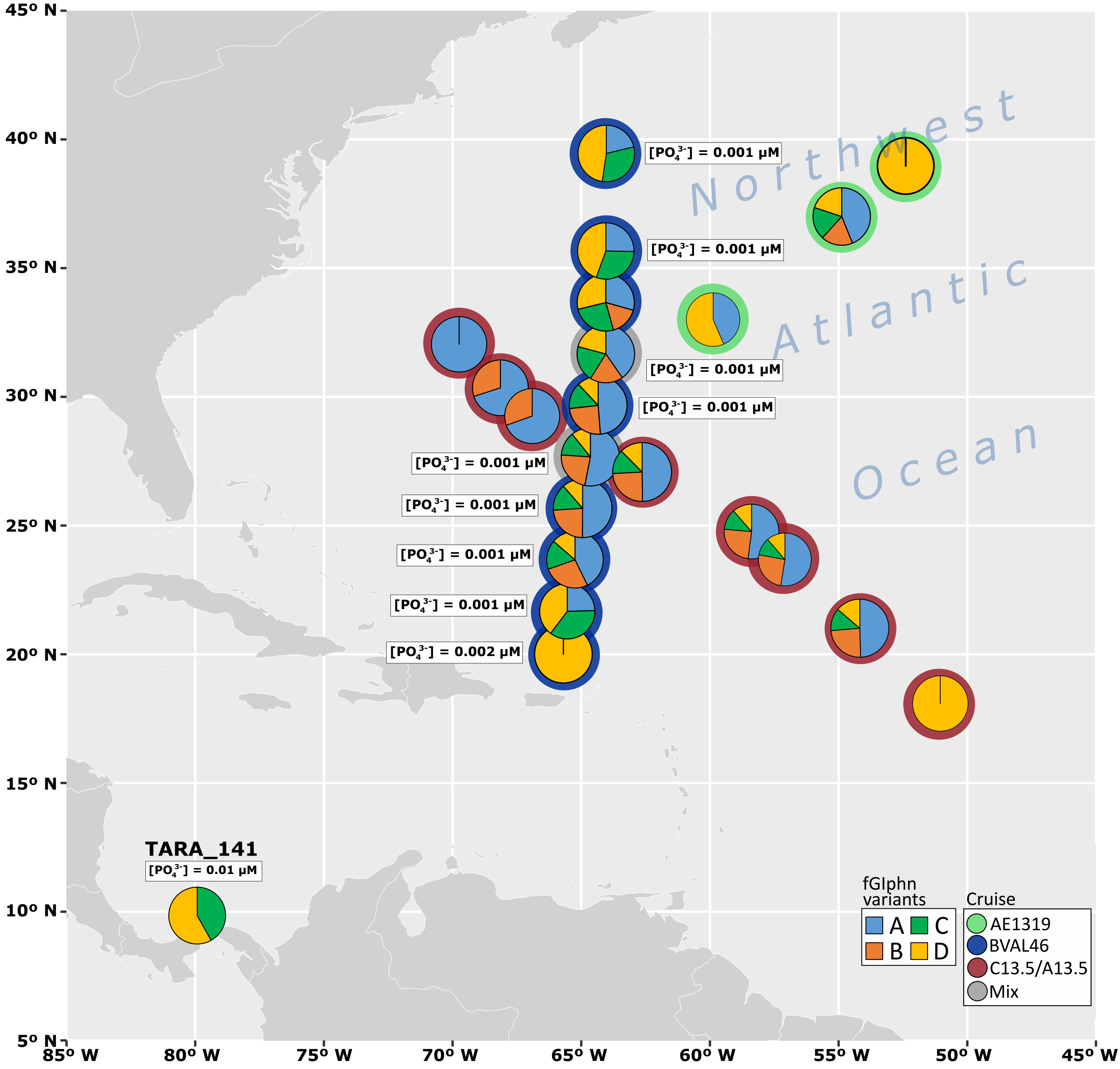

Figure S3. Relative abundance of each version of the flexible phosphonate-related genomic island (fGIphn) in North Atlantic Ocean samples from the Tara Oceans and Bio-GO-SHIP expeditions. Phosphate concentration is shown for those stations where it was determined. The different cruises of the Bio-GO-SHIP expedition are highlighted with different coloured circles.
