# Supplementary material for: Phosphate-related genomic islands as drivers of environmental adaptation in the streamlined marine alphaproteobacterial HIMB59
Source: mSystems. 2023 Dec 6;8(6):e00898-23. doi: 10.1128/msystems.00898-23 (PMC10734472; doi:10.1128/msystems.00898-23)
Supplement: Supplemental Information — Supplemental text, Fig. SI-1, and Tables SI-1 to SI-3. [file msystems.00898-23-s0004.pdf]

Phosphate-related genomic islands as drivers of environmental adaptation in the streamlined marine alphaproteobacterial HIMB59

Carmen Molina-Pardines[a,b], Jose M. Haro-Moreno[a,b] and Mario López-Pérez[a*]

[a]Evolutionary Genomics Group, División de Microbiología, Universidad Miguel Hernández, Apartado 18, San Juan 03550, Alicante, Spain.

[b]These authors contributed equally to this work

*Corresponding author: mario.lopezp@umh.es

Evolutionary Genomics Group, División de Microbiología, Universidad Miguel Hernández, Apartado 18, San Juan de Alicante, 03550 Alicante, Spain.

Phone +34-965919313, Fax +34-965 919457

Comparative genomics among genomospecies of the order HIMB59

We used a genomic approach to compare the overall gene content of the three major genomospecies of the order HIMB59 by clustering genes from all genomes and then removing the common part between them. The set of specific genes was analyzed at the functional level using the SEED Subsystems database (1). Genomospecies GCA002718135-1.A showed an enrichment in oligopeptide-related ABC transporters as well as in the transport and utilization of several monosaccharides (L-fucose, L-rhamnose, L-arabinose, Xylose, D-ribose and Myo-inositol). Although all three genomospecies had the Leloir pathway, in genomospecies GCA002718135-1.A we also found the alternative DeLey-Doudoroff pathway. These two pathways are related to the metabolism and utilization of galactose as a source of energy and carbon (2, 3). Given that both metabolic pathways were absent in the order *Pelagibacteriales* (SAR11), they could represent a survival strategy to avoid competition for the same resources in the same niche. GCA002718135-1.A also showed genomic potential for the transport and catabolism of urea that can be used as a nitrogen source.

To evaluate in detail this enrichment in certain metabolic functions of GCA002718135-1.A, we used three specific databases to analyse the presence of glycoside hydrolases, peptidases as well as transporters such as the CAZY (Table SI1), MEROPS (Table SI2) and KEGG (Table SI3), respectively (4–6). The results obtained for the reference genomes of each genomospecies, described in the main text, were compared with the data obtained against a reference collection of pelagic marine microbes with different ecological functions by normalizing the values by megabase of genome (Figure SI1). The analysis revealed that genomospecies GCA002718135-1.A had the largest number of GHs/Mb of the three HIMB59 genomospecies, including the pure culture (Figure SI1 and Table SI1). Notably, this genomospecies (GCA002718135-1.A) also showed the highest values among the marine reference microbes including *Polaribacter* sp. MED152, a free-living marine bacterium of the phylum Bacteroidetes characterized as a specialist in degrading polymeric carbohydrates (7) (Figure SI1 and

Table SI1). While *Polaribacter* sp. MED152 and the other reference aerobic heterotrophic bacterium, *Alteromonas macleodii* AD45, showed the highest diversity of GHs, in the reference genome of GCA002718135-1.A we found the predominance of GH109, a family of enzymes with α-N-acetylgalactosaminidase activity (Figure SI1 and Table SI1). This hydrolase catalyzes the removal of both terminal α-N-acetylgalactosamine residues and galactose monosaccharides from glycoconjugates or complex polysaccharides. This result together with the presence of the Leloir and DeLey-Doudoroff pathways (see above) suggest an important role of galactose metabolism in members of this family. Regarding peptidase prediction based on MEROPS classification, HIMB59 genomospecies showed the same proportion, ranging from 35 to 39 proteins per Mb (Figure SI1). The most abundant families were cysteine, serine and metallo peptidases. GCA002718135-1.A had a higher proportion of metallopeptidases, whilst genomospecies HIMB59-1.A and 1.B were enriched in serine peptidases (Figure SI1 and Table SI2). *A. macleodii* AD45 had the highest proportion of peptidases of all genomes, possibly due to its copiotrophic nature. The abundance of genes related to membrane transport showed that genomes of the order HIMB59 had the highest values of all genomes analyzed according to the KEGG database. We found some differences in terms of abundance concerning specific nutrient acquisition. Among all the genomes analyzed, the genomospecies GCA002718135-1.A had the highest proportion of proteins potentially involved in the transport of saccharide, polyol, and lipids; HIMB59-1.A the transport of phosphate and organophosphate molecules; whereas the pure culture had a higher fraction of mineral and organic ion transporters (Figure SI1 and Table SI3).

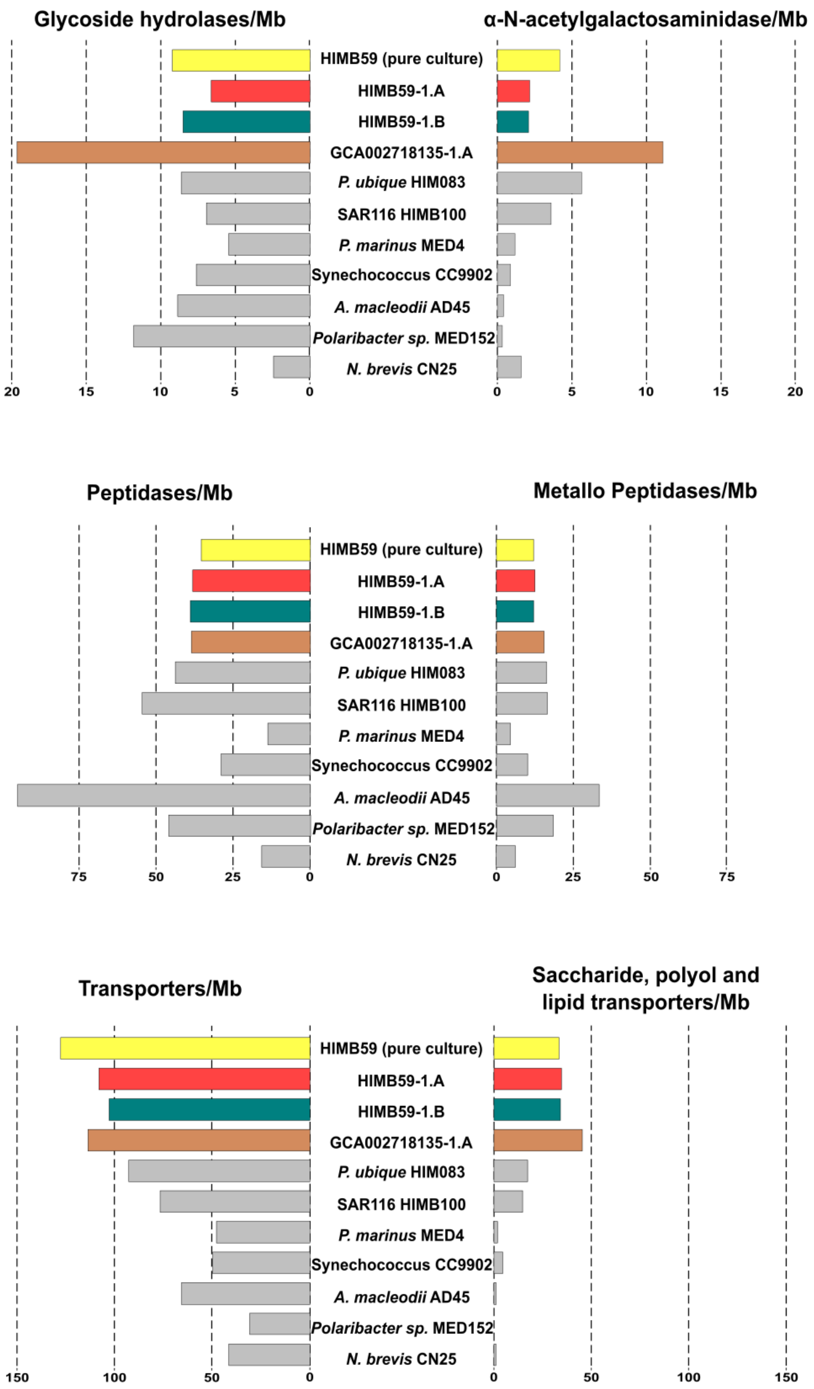

Figure SI-1. Number of genes normalized per megabase of genome for each of the functional categories. The coloured bars indicate the origin of the genome i. e pure culture (yellow), HIMB59-1.A genomospecies (red), HIMB59-1.B genomospecies (green), GCA002718135-1.A (light brown) and representative marine microbe genomes (gray).

Table SI-1. Abundance of genes affiliated with glycoside hydrolase (GH) families based on the CAZY database

	HIMB59 order				Reference marine microbes						
Category	HIMB59 (pure culture)	HIMB59-1.A	HIMB59-1.B	GCA002718135-1.A	*Pelagibacter ubique* HIMB083	SAR116 HIMB100	*Prochlorococcus marinus* MED4	*Synechococcus* CC9902	*Alteromonas macleodii* AD45	*Polaribacter* sp. MED152	*Nitrosopelagicus brevis* CN25
GH1	0.71	0.74	0.71	0.00	0.00	0.41	0.00	0.00	0.22	0.00	0.00
GH2	0.00	0.00	0.00	0.60	0.00	0.00	0.00	0.00	0.43	0.34	0.00
GH3	0.71	0.00	0.71	0.00	0.72	0.00	0.00	0.45	0.43	0.00	0.00
GH4	0.71	0.74	1.42	1.19	0.00	0.41	0.00	0.00	0.00	0.00	0.00
GH5	0.00	0.00	0.00	0.00	0.00	0.00	0.00	0.00	0.00	0.68	0.00
GH8	0.00	0.00	0.00	0.00	0.00	0.00	0.00	0.00	0.22	0.00	0.00
GH13	0.71	0.74	0.71	0.00	0.00	0.41	1.82	2.24	2.16	2.70	0.00
GH15	0.00	0.00	0.00	0.00	0.00	0.00	0.00	0.45	0.00	0.00	0.00
GH16	0.00	0.00	0.00	0.00	0.00	0.00	0.00	0.00	0.22	0.68	0.00
GH17	0.00	0.00	0.00	0.00	0.00	0.00	0.00	0.00	0.00	0.34	0.00
GH20	0.00	0.00	0.00	0.60	0.00	0.00	0.00	0.00	0.22	0.68	0.00
GH23	0.71	0.74	0.71	0.60	0.72	0.00	0.00	0.00	0.43	0.68	0.00
GH24	0.00	0.00	0.00	0.60	0.00	0.00	0.00	0.00	0.00	0.00	0.00
GH28	0.00	0.00	0.00	0.00	0.00	0.00	0.00	0.00	0.43	0.00	0.00
GH29	0.00	0.00	0.00	0.60	0.00	0.00	0.00	0.00	0.00	0.00	0.00
GH30	0.00	0.00	0.00	0.00	0.00	0.00	0.00	0.00	0.00	0.68	0.00
GH31	0.00	0.00	0.00	0.00	0.00	0.00	0.00	0.00	0.43	0.68	0.00
GH32	0.00	0.00	0.00	0.60	0.00	0.00	0.00	0.00	0.00	0.00	0.00
GH33	0.00	0.00	0.00	0.60	0.00	0.00	0.61	0.00	0.00	0.00	0.81
GH36	0.00	0.00	0.00	0.00	0.00	0.00	0.00	0.00	0.22	0.00	0.00
GH37	0.00	0.00	0.00	0.00	0.00	0.00	0.00	0.00	0.22	0.34	0.00
GH42	0.00	0.00	0.71	0.00	0.00	0.41	0.00	0.00	0.00	0.00	0.00
GH51	0.00	0.00	0.00	0.60	0.00	0.00	0.00	0.00	0.00	0.00	0.00
GH53	0.00	0.00	0.00	0.00	0.00	0.00	0.00	0.00	0.00	0.34	0.00
GH57	0.00	0.00	0.00	0.00	0.00	0.00	0.61	0.90	0.00	0.00	0.00
GH63	0.00	0.00	0.00	0.00	0.00	0.41	0.00	0.00	0.00	0.00	0.00
GH65	0.00	0.00	0.00	0.00	0.00	0.00	0.00	0.00	0.00	0.68	0.00
GH73	0.71	0.74	0.71	0.60	0.72	0.41	0.00	0.00	0.43	0.34	0.00
GH74	0.00	0.00	0.00	0.00	0.00	0.00	0.00	0.45	0.00	0.34	0.00
GH76	0.00	0.00	0.00	0.00	0.00	0.00	0.00	0.00	0.22	0.00	0.00
GH77	0.00	0.00	0.00	0.00	0.00	0.00	0.61	0.45	0.22	0.00	0.00
GH81	0.00	0.00	0.00	0.00	0.00	0.00	0.00	0.00	0.00	0.34	0.00
GH84	0.00	0.00	0.00	0.00	0.00	0.41	0.00	0.00	0.00	0.00	0.00
GH85	0.00	0.00	0.00	0.00	0.00	0.00	0.00	0.00	0.22	0.00	0.00
GH92	0.00	0.00	0.00	0.00	0.00	0.00	0.00	0.00	0.87	0.34	0.00
GH93	0.00	0.00	0.00	0.60	0.00	0.00	0.00	0.00	0.00	0.00	0.00
GH100	0.00	0.00	0.00	0.00	0.00	0.00	0.61	0.45	0.00	0.00	0.00
GH103	0.71	0.74	0.71	0.60	0.72	0.41	0.00	0.00	0.43	0.00	0.00
GH104	0.00	0.00	0.00	0.00	0.00	0.00	0.00	0.90	0.00	0.00	0.00
GH105	0.00	0.00	0.00	0.00	0.00	0.00	0.00	0.00	0.22	0.00	0.00
GH109	4.26	2.21	2.13	11.31	5.76	3.67	1.21	0.90	0.43	0.34	1.63
GH113	0.00	0.00	0.00	0.00	0.00	0.00	0.00	0.00	0.00	0.34	0.00
GH116	0.00	0.00	0.00	0.00	0.00	0.00	0.00	0.45	0.00	0.00	0.00
GH127	0.00	0.00	0.00	0.60	0.00	0.00	0.00	0.00	0.00	0.00	0.00
GH149	0.00	0.00	0.00	0.00	0.00	0.00	0.00	0.00	0.00	0.34	0.00
GH163	0.00	0.00	0.00	0.00	0.00	0.00	0.00	0.00	0.00	0.34	0.00
GH171	0.00	0.00	0.00	0.00	0.00	0.00	0.00	0.00	0.22	0.34	0.00
**Total GH/Mb**	**9.22**	**6.62**	**8.51**	**19.64**	**8.63**	**6.94**	**5.45**	**7.62**	**8.87**	**11.82**	**2.44**

Table SI-2. Abundance of genes affiliated with peptidases based on the MEROPS database.

	HIMB59 order				Reference marine microbes						
Catalytic type peptidase	HIMB59 (pure culture)	HIMB59-1.A	HIMB59-1.B	GCA002718135-1.A	*Pelagibacter ubique* HIMB083	SAR116 HIMB100	*Prochlorococcus marinus* MED4	*Synechococcus* CC9902	*Alteromonas macleodii* AD45	*Polaribacter* sp. MED152	*Nitrosopelagicus brevis* CN25
Aspartic (A) Peptidases	0.71	0.74	0.71	0.60	1.63	0.72	0.22	1.01	2.42	0.82	0.00
Cysteine (C) Peptidases	7.80	6.62	8.51	5.95	7.32	7.91	1.52	3.72	7.88	2.45	3.14
Metallo (M) Peptidases	12.06	12.50	12.06	15.48	16.26	16.55	4.55	10.14	33.33	18.37	6.28
Non-peptidase homologue	2.13	1.47	2.13	3.57	3.25	2.88	0.87	1.35	3.64	1.22	1.35
Peptidase inhibitors	1.42	1.47	1.42	1.19	1.63	0.72	0.22	0.68	1.82	2.04	0.90
Unknown catalytic type	0.00	0.00	0.00	0.00	0.00	0.72	0.00	0.00	0.61	0.00	0.00
Serine (S) Peptidases	9.93	13.24	12.06	10.12	13.01	22.30	6.28	11.49	39.39	20.00	2.24
Threonine (T) Peptidases	1.42	2.21	2.13	1.79	0.81	1.44	0.22	0.68	4.85	1.22	1.79
**Total Peptidases/Mb**	**35.46**	**38.24**	**39.01**	**38.69**	**43.90**	**54.68**	**13.85**	**29.05**	**95.15**	**46.12**	**15.70**

Table SI-3. Abundance of genes affiliated with membrane transport function based on the KEGG database.

	HIMB59 order				Reference marine microbes						
**Subfamily**	**HIMB59 (pure culture)**	**HIMB59-1.A**	**HIMB59-1.B**	**GCA002718135-1.A**	***Pelagibacter ubique* HIMB083**	**SAR116 HIMB100**	***Prochlorococcus marinus* MED4**	***Synechococcus* CC9902**	***Alteromonas macleodii* AD45**	***Polaribacter* sp. MED152**	***Nitrosopelagicus brevis* CN25**
ABC-2 type and others	8.51	5.15	7.80	7.14	7.91	6.12	6.06	6.28	6.71	7.09	5.69
ABCB (MDR/TAP) subfamily	1.42	0.74	1.42	1.79	2.16	1.63	2.42	3.14	1.08	1.01	0.00
ABCC (CFTR/MRP) subfamily	0.00	0.00	0.00	0.00	0.00	0.00	0.00	0.00	0.43	0.00	0.00
Accessory factors involved in transport	0.00	0.00	0.00	0.00	0.00	0.41	0.00	0.90	1.95	2.70	0.00
Aquaporins and small neutral solute transporters	0.00	0.00	0.00	0.00	0.00	0.00	0.00	0.00	0.22	0.00	1.63
Drug transporters	0.00	0.00	0.00	0.00	0.00	0.41	0.61	0.45	0.87	0.00	0.00
Electrochemical potential-driven transporters	19.15	14.71	14.89	8.33	16.55	11.84	7.27	6.28	12.12	7.09	4.07
Phosphotransferase system (PTS)	0.00	0.00	0.00	0.00	0.00	0.00	0.00	0.00	0.65	0.00	0.00
Metal transporters	0.00	0.00	0.00	0.00	0.00	0.00	0.00	0.45	0.00	0.00	0.00
Iron-siderophore and vit B12 transporters	2.84	3.68	3.55	2.38	2.88	2.04	6.06	4.48	0.65	1.01	3.25
Mineral and organic ion transporters	24.82	13.24	12.77	8.93	12.95	9.80	3.64	1.35	3.03	0.00	2.44
Nitrate/nitrite transporters	0.00	0.00	0.00	0.00	0.00	0.00	0.00	0.45	0.00	0.00	0.00
Organic acid transporters	0.00	0.00	0.00	0.00	0.00	0.00	0.00	0.00	0.43	0.34	0.00
Others	0.71	0.74	0.71	0.60	0.72	0.82	0.61	1.35	1.73	1.69	1.63
Peptide and nickel transporters	6.38	0.00	0.00	16.67	3.60	4.08	3.03	2.24	2.16	0.00	4.07
Phosphate and organophosphate transporters	14.89	20.59	11.35	7.74	14.39	11.02	8.48	7.62	2.16	0.00	0.00
Pores ion channels	11.35	9.56	9.93	10.71	8.63	10.20	7.27	9.87	26.41	16.55	4.88
Primary active transporters	3.55	4.41	4.96	3.57	4.32	2.45	0.61	0.45	1.73	1.01	2.44
Protein transporters	0.71	0.74	0.71	0.00	0.72	0.41	0.00	0.00	0.22	0.00	0.00
Saccharide, polyol, and lipid transporters	33.33	34.56	34.04	45.24	17.27	14.69	1.82	4.48	1.08	1.01	0.00
Solute carrier family (SLC)	0.00	0.00	0.71	0.60	0.72	0.82	0.00	0.00	0.87	1.01	0.81
Sugar transporters	0.00	0.00	0.00	0.00	0.00	0.00	0.00	0.00	0.22	0.00	0.00
**Total transporters/Mb**	**127.66**	**108.09**	**102.84**	**113.69**	**92.81**	**76.73**	**47.88**	**49.78**	**65.80**	**41.55**	**30.89**

References

1. Overbeek R, Begley T, Butler RM, Choudhuri J V., Chuang HY, Cohoon M, de Crécy-Lagard V, Diaz N, Disz T, Edwards R, Fonstein M, Frank ED, Gerdes S, Glass EM, Goesmann A, Hanson A, Iwata-Reuyl D, Jensen R, Jamshidi N, Krause L, Kubal M, Larsen N, Linke B, McHardy AC, Meyer F, Neuweger H, Olsen G, Olson R, Osterman A, Portnoy V, Pusch GD, Rodionov DA, Rückert C, Steiner J, Stevens R, Thiele I, Vassieva O, Ye Y, Zagnitko O, Vonstein V. 2005. The subsystems approach to genome annotation and its use in the project to annotate 1000 genomes. Nucleic Acids Res 33:5691–5702.

2. Wong TY, Yao XT. 1994. The DeLey-Doudoroff Pathway of Galactose Metabolism in Azotobacter vinelandii. Appl Environ Microbiol 60:2065–2068.

3. Tästensen JB, Johnsen U, Reinhardt A, Orthjohann M, Schönheit P. 2020. D-galactose catabolism in archaea: operation of the DeLey–Doudoroff pathway in Haloferax volcanii. FEMS Microbiol Lett 367:29.

4. Barrett AJ, Rawlings ND, O'brien EA. 2001. The MEROPS database as a protease information system. J Struct Biol 134:95–102.

5. Kanehisa M, Sato Y, Kawashima M, Furumichi M, Tanabe M. 2016. KEGG as a reference resource for gene and protein annotation. Nucleic Acids Res 44:D457–D462.

6. Lombard V, Golaconda Ramulu H, Drula E, Coutinho PM, Henrissat B. 2014. The carbohydrate-active enzymes database (CAZy) in 2013. Nucleic Acids Res 42.

7. McKee LS, La Rosa SL, Westereng B, Eijsink VG, Pope PB, Larsbrink J. 2021. Polysaccharide degradation by the Bacteroidetes: mechanisms and nomenclature. Environ Microbiol Rep 13:559–581.
